# Report of a Case of Syphilis Cured by One Dose of Salvarsan with Reinfection Two Years Later

**Published:** 1916-10

**Authors:** Chas. W. Bethune

**Affiliations:** Buffalo, N. Y.


### Report of a Case of Syphilis Cured by One Dose of Salvarsan With Reinfection Two Years Later.

CHAS. W. BETHUNE, M. D., Buffalo, N. Y.

L. P., bartender, was admitted to the Sisters' Hospital Dec. 1913, giving a history of syphilitic infection eight months previously, penile chancre, roseola, loose hair and positive Wassermann. This diagnosis having been made by a practitioner in an adjoining town. He later visited a quack who claimed to administer Neosalvarsan, but there is strong evidence to prove that sodium cacodyalite was the drug administered.

The patient presented no visible signs of syphilis except a general adenitis. Dr. Bentz reported a three plus Wassermann.

0.60 Salvarsan was administered intravenously. The patient then left the hospital declining further treatment. He reported at my office one year later to be treated for gonorrhoea, but declined further treatment with Salvarsan, claiming that the Health Department had found his Wassermann negative.

May 9, 1916, he reported at my office with a chancre of the tonsil, adenitis and roseola, Wassermann two plus (Board of Health).

Dr. Grover Wende kindly confirmed my diagnosis of a recent infection of syphilis. The patient permitted me to administer two doses of 0.90 Neosalvarsan and then refused further treatment. The visible lesions disappearing in a couple of days.

Cases of reinfection of syphilis after Salvarsan treatment are frequently reported, but the first infection was either seen early or if old was thoroughly treated. In this case an infection of eight months duration was apparently cured by one dose of Salvarsan.

294 South Elmwood.

---

**Military Deaf-Mutism From Commotion.** L. Lortat-Jacob and J. B. Buvat, Soc. Med. des Hop., Feb. 11, report that this condition has been found in numerous cases unattached by aural lesion and they consider it hysteric. In 7 cases, the distance from the explosion was great enough to leave in doubt whether concussion was a factor at all. Six of these cases were cured by ipecac, in emetic dose. They do not decide whether the result depended on any direct reflex connection of the glosso-pharyngeal and the auditory nerves or whether it was entirely suggestive.

